# Supplementary material for: MetaRibo-Seq measures translation in microbiomes
Source: Nat Commun. 2020 Jun 29;11:3268. doi: 10.1038/s41467-020-17081-z (PMC7324362; doi:10.1038/s41467-020-17081-z)
Supplement: Supplementary file 10 — Supplementary Data 7 [file 41467_2020_17081_MOESM10_ESM.zip › File2/Confidence_High_Taxonomy/71118_out.krona.html]

Javascript must be enabled to view this page.

members
magnitude
magnitudeUnassigned
count
unassigned
taxon
rank

71118\_out

2

2
2
superkingdom

2
phylum
201174

2
class
1760

85007
2
order

family
2
1653

1716
genus
2

61592

SRS017814\_contig\_number\_8192SRS065099\_contig\_number\_contig-100\_2169.147077
2
species
